# Reduced neural investment in post-reproductive females of the bee *Ceratina calcarta*

**DOI:** 10.1038/s41598-022-12281-7

**Published:** 2022-05-18

**Authors:** Sarah Jaumann, Sandra M. Rehan, Kayla Schwartz, Adam R. Smith

**Affiliations:** 1 Department of Biological Sciences, George Washington University, 800 22nd St. NW, Washington, DC 20052 USA; 2 Department of Biology, York University, Toronto, ON Canada

**Keywords:** Social behaviour, Social evolution

## Abstract

Many insects show plasticity in the area of the brain called the mushroom bodies (MB) with foraging and social experience. MBs are paired neuropils associated with learning and memory. MB volume is typically greater in mature foragers relative to young and/or inexperienced individuals. Long-term studies show that extended experience may further increase MB volume, but long-term studies have only been performed on non-reproductive social insect workers. Here we use the subsocial bee *Ceratina calcarata* to test the effect of extended foraging experience on MB volume among reproductive females. *Ceratina calcarata* females forage to provision their immature offspring in the spring, and then again to provision their adult daughters in the late summer. We measured the volume of the MB calyces and peduncle, antennal lobes (AL), optic lobes (OL), central complex (CX), and whole brains of three groups of bees: newly emerged females, reproductive females in spring (foundresses), and post-reproductive mothers feeding their adult daughters in late summer. Post-reproductive late summer mothers had smaller MB calyces and ALs than foundresses. Moreover, among late mothers (but not other bees), wing wear, which is a measure of foraging experience, negatively correlated with both MB and OL volume. This is contrary to previously studied non-reproductive social insect workers in which foraging experience correlates postiviely with MB volume, and suggests that post-reproductive bees may reduce neural investment near the end of their lives.

## Introduction

Adult brain plasticity in response to accumulated experience or environmental change is widespread among animals. In insects, plasticity in response to adult experience is especially prominent in the mushroom body (MB)^1,2^. The mushroom bodies are paired neuropils that support higher-order cognitive processes such as sensory integration, learning and memory^2,3^. Previous studies have shown expansion in the MBs of adult insects resulting from foraging experience, social interactions, and age, termed ‘experience dependant plasticity’^1^.

MBs may increase in volume in response to foraging experience, especially central-place foraging, which requires spatial memory and navigation^4–20^. Typically, this increase in MB volume is demonstrated by comparing young or inexperienced individuals with experienced foragers^7,15^ or by comparing an experimentally restricted group with one allowed to forage^11,19,20^.

Another type of adult experience that can lead to increased MB volume is social experience. *Drosophila* housed in groups had larger MBs than those reared alone^21^. In the ant *Camponotus floridanus,* socially isolated individuals had smaller MB calyces than ants in a social nest^22^. The solitary bee *Nomia melanderi* showed increased growth in the lip region of the MB calyx when experimentally paired with another bee^23^. In some species of bees and paper wasps social dominance of reproductive queens over subordinate workers, rather than social interactions themselves, result in increased MB calyx volume of social dominants relative to subordinates^12,15,24–26^. However, this may be a result of reduced MB calyx volume in workers, rather than, or in addition to, increased volume in queens^15,27^, or a result of different developmental pathways rather than adult plasticity^28^. Rehan et al.^15^ used the facultatively social and solitary bee *Ceratina australensis* to show that foraging experience (in both social and solitary reproductives) and social dominance (in the social ones) each contributed to increased MB calyx volume.

Lastly, MB volume may increase with age in early adulthood before the onset of foraging. Workers of honeybees increase MB neuropil volume immediately following adult emergence, but before foraging (this is termed ‘experience-expectant’ plasticity)^5,18,20,29^. Other species with similar ‘experience-expectant’ increases in MB volume in early adulthood include bumblebees^30^, paper wasps^25^, and some ants^7,16,22^. These are all species that live in social groups where young workers do not need to begin foraging immediately. Data are mixed on whether experience-expectant plasticity occurs in the facultatively social and solitary sweat bee *Megalopta genalis*^27,31^, but experience-expectant plasticity was not found in the only two species of solitary bees in which it was investigated^19,23^.

Studies that follow insects into 'old age' (well beyond the onset of foraging) have been conducted on honeybee and ant workers, where individuals accumulate experience, but do not reproduce. These studies show incremental increases in MB volume in ants^7,10,17,32^, and no effect of extended foraging experience on MB volume in honeybees^11,33^. No study has examined the effect of extended foraging through and beyond the reproductive period in a solitary or small-colony insect.

Here we use the small carpenter bee *Ceratina calcarata* to test for an effect of extended age, experience, and social interactions on brain development. *Ceratina calcarata* mothers engage in two distinct rounds of foraging. First, in spring they initiate a nest and provision their offspring with pollen and nectar, similarly to most other bees (this is the reproductive foundress stage). When their daughters emerge in late summer, the mothers undertake a second round of foraging to further provision their now adult daughters; this is atypical among bees (this is the late mother stage)^34,35^. The adult daughters will enter diapause during the fall and winter, and emerge the following spring to initiate their own nests. Mothers forage at the same rate as their daughters, and the second bout of foraging from the late mothers increases overwintering success of the daughters. The late mothers die soon after they cease foraging^35^.

The life history of *C. calcarata* results in three groups of bees of increasing age and foraging experience: newly emerged daughters, reproductive foundresses, and late mothers. Based on previous studies of adult brain plasticity, reproductive foundresses should have greater MB calyx volume than newly emerged daughters because they have foraging experience and are older. Likewise, late mothers should have greater MB calyx volume than reproductive foundresses because they have even more foraging experience, are older still, and have social interactions with their adult daughters. The maternal care of late mothers is different than the social dominance of *C. australensis* or other social bees and wasps cited above because the mother is supporting her daughter, who will be a reproductive female the following spring, rather than supressing reproduction or establishing dominance^15^.

In order to examine brain development with increasing age and experience in *C. calcarata,* we measured the MB calyces and MB peduncle, as well as the optic lobes (OL), antennal lobes (AL), and central complex (CX) of the brain. We measured the MB calyx because this was the area of the brain affected by foraging and social dominance in another species of *Ceratina* bee, *C. australensis*^15^. We measured the OL and AL because these areas may respond to increased visual and chemical stimulation, respectively, associated with foraging and social experience (e.g.^36–38^). And we measured the CX because it is also involved in learning and sensory integration^39^. In addition to the brain areas, we measured ovary size to investigate the effect of reproductive status on brain area volume, and also wing wear, which correlates with flight activity, as another measure of foraging experience^15^. We predicted that the MB calyx, OL, and AL would all increase with age and experience. An alternative prediction is that if females reduce the expense of maintaining neural tissue after reproduction, late mothers would have reduced MB calyx, OL, and AL volume relative to reproductive foundresses.

## Methods

### Collections

Females were collected from nests of staghorn sumac (*Rhus typhina*) in and around Durham, NH in May–August 2018. Nest entrances were covered with masking tape and the base of the stem cut with pruning shears to remove dead stems from the trees. Nests were stored at 4 °C until dissection the same day in the lab. Adult females were placed in 4% paraformaldehyde in phosphate-buffered saline (PBS) at collection and stored them at 4 °C until dissection. Females establish nests in late spring (May) and forage to provision nests (June) followed by guarding developing brood (July) and interacting with adult offspring (August)^34^. Foundresses were collected in May of 2018. Late summer mothers and recently emerged late summer daughters were collected in August of 2018.

### Brain volume measurements

**Figure 1:**
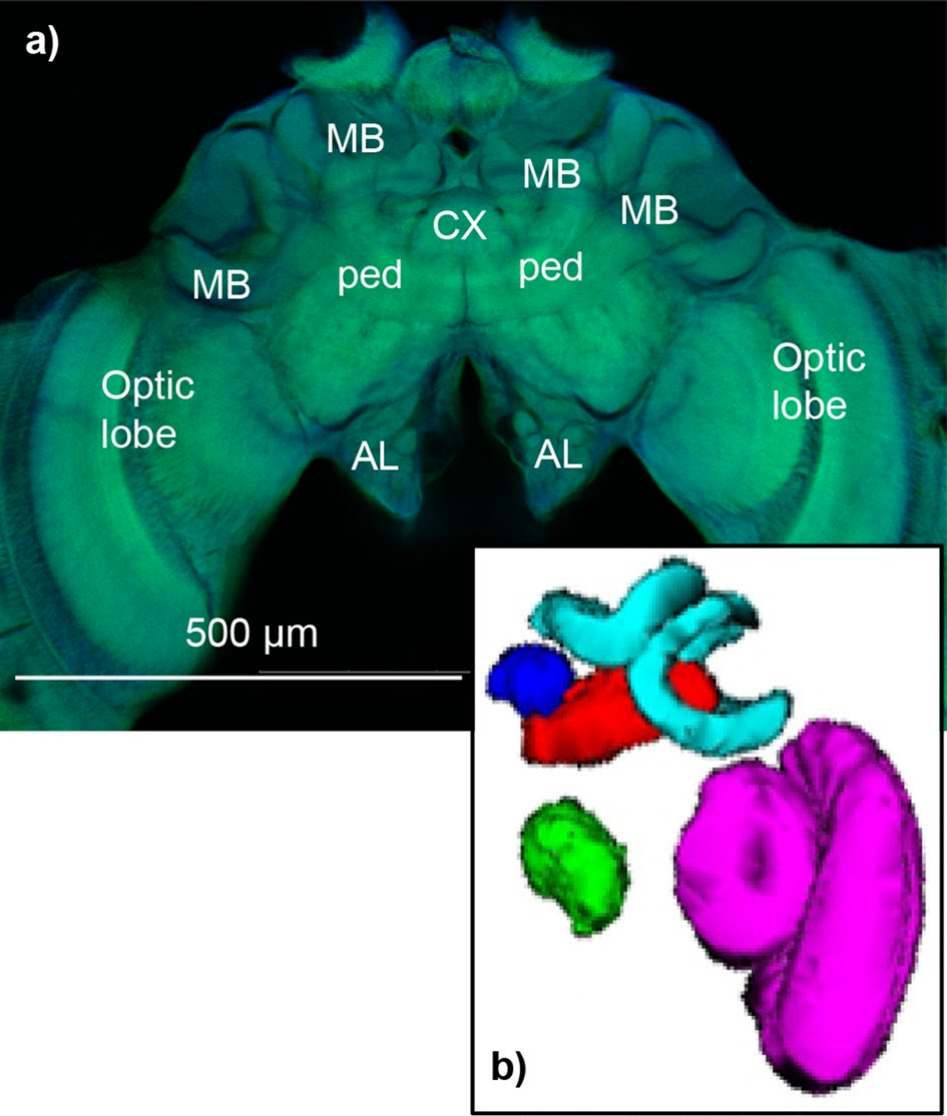
(**a**) Confocal micrograph showing mushroom body calyces (MB) and peduncle (ped), the central complex (CX), antennal lobes (AL), and optic lobes; scale bar = 500 μm. (**b**) Reconstructed volume of a representative brain, with AL in green, CX in royal blue, MB peduncle in red, MB calyces in light blue, and OL in pink.

We dissected head capsules in PBS from seven females in each group (21 total) to remove the brain which was immediately placed in glutaraldehyde (2%) for 48 h, bleached in a formamide solution, and dehydrated in a series of ethanol washes of increasing concentration following^40^. Prior to imaging, brains were mounted in methyl salicylate. Brains were imaged using an Olympus Fluoview FV1000 confocal microscope using autofluorescence at 10X magnification and a step size of 10 μm (Fig. 1a). We calculated volumes of the whole brain (excluding the subesophegeal ganglion) and different neuropils through tracing and serial reconstruction using the software program Reconstruct^41^ (Fig. 1b). We measured the MB calyces, MB peduncle, AL, and OL (medulla + lobula) of one side of the brain and multiplied the resulting volumes by two to calculate total volume. Likewise, we measured the right hemisphere and multiplied by two to calculate whole brain volume. We measured the right side of the brain (viewed frontally) unless that side was damaged. We measured the CX, including the ellipsoid body, the superior arch, and the fan shaped body, but not the paired noduli or protocerebral bridge. Brain and neuropil volumes were standardized to average body size (head width was our measure of body size) by calculating a correction factor that was applied to each bee: mean body size of all bees in the study divided by the individual’s body size. This correction factor was then multiplied to brain and neuropil volume for each bee.

### Physical measurements

We used head width as a measure of body size. Wing wear was scored on a scale of 0–5 following^15,42^ in which zero represents bees with unworn forewing margins, and 5 represents bees with none of the origninal forewing margin remaining and wear extending to the wing veins. Wings were examined under a dissecting microscope at 10 × magnification for scoring. Ovary development was scored on a scale of 1–5 following^43^ in which one represents ovaries with no visible developing oocytes and five represents ovaries with nearly fully developed eggs. Ovaries were exposed by removing the abdominal tergites and gut at 10 × magnification under a dissecting light microscope. Scores for left and right forewings and left and right ovaries, respectively, were averaged for each individual bee.

### Stastical analyses

Wing wear scores and ovary ratings were analyzed with non-parametric statistics (Kruskal–Wallis and Spearman’s rank correlations) because they are ordinal variables. Head width and volumes of brain areas were compared between groups using ANOVA followed by Tukey’s post-hoc comparisons. Antennal lobe volume and central complex volume were both log-transformed to fit the assumption of normality.

## Results

### Physical measures

**Figure 2:**
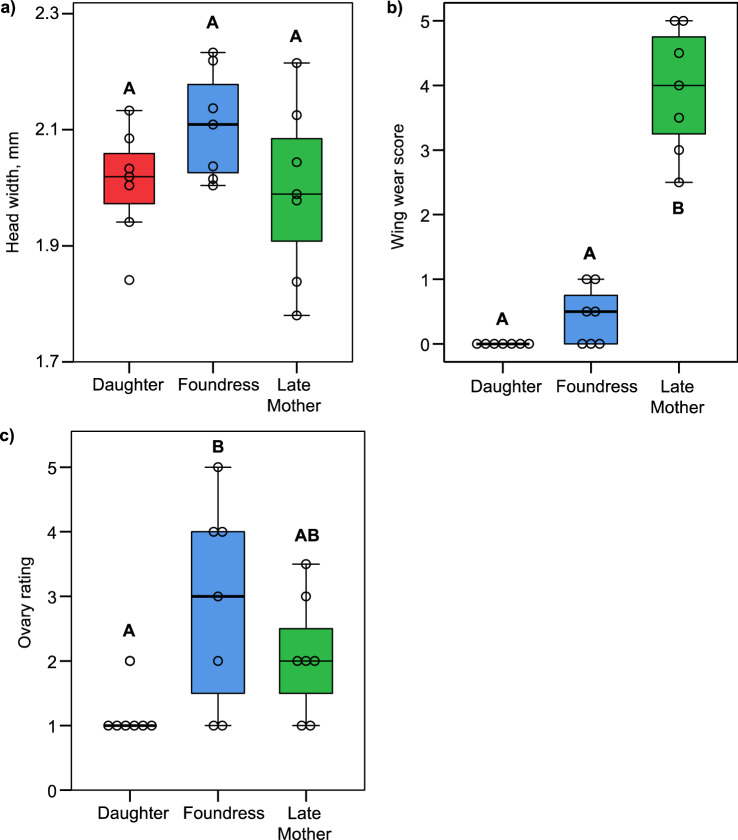
Boxplots showing head width, a measure of body size (**a**), wing wear (**b**) and ovary development (**c**) among newly emerged daughters, foundresses, and late mothers. N = 7 for each group. Note that no newly emerged daugthers exhibited wing wear, and only one exhibited ovarian development. Groups that do not share a letter are significantly different from each other based on ANOVA (**a**) or Kruskil-Wallis test followed by Bonferroni-corrected pairwise comparisons (**b**,**c**).

There were no differences in body size, measured as head width, between groups (*F*_2,18_ = 1.929, *p* = 0.174; Fig. 2a). Late mothers had more worn wings than reproductive foundresses and newly emerged daughters; newly emerged daughters showed no wing wear at all (Kruskal–Wallis test = 16.633, df = 2, p < 0.001; Bonferroni corrected post-hoc p values = 0.020 and p < 0.001, respectively, Fig. 2b). Foundresses and newly emerged daughters did not differ in degree of wing wear (p = 0.604; Fig. 2b).

Ovary size increased from the newly emerged daughter to reproductive foundress stage (Kruskal–Wallis test = 6.629, df = 2, p = 0.036; Fig. 2c). Foundresses had significantly larger ovaries than newly emerged daughters, but not late mothers (Bonferroni corrected post-hoc p values = 0.037 and p = 1.00, respectively). Newly emerged daughters and late mothers did not differ (p = 1.00; Fig. 2c). Only one of the newly emerged daugthers showed any ovary development.

### Neurobiological measures

**Figure 3:**
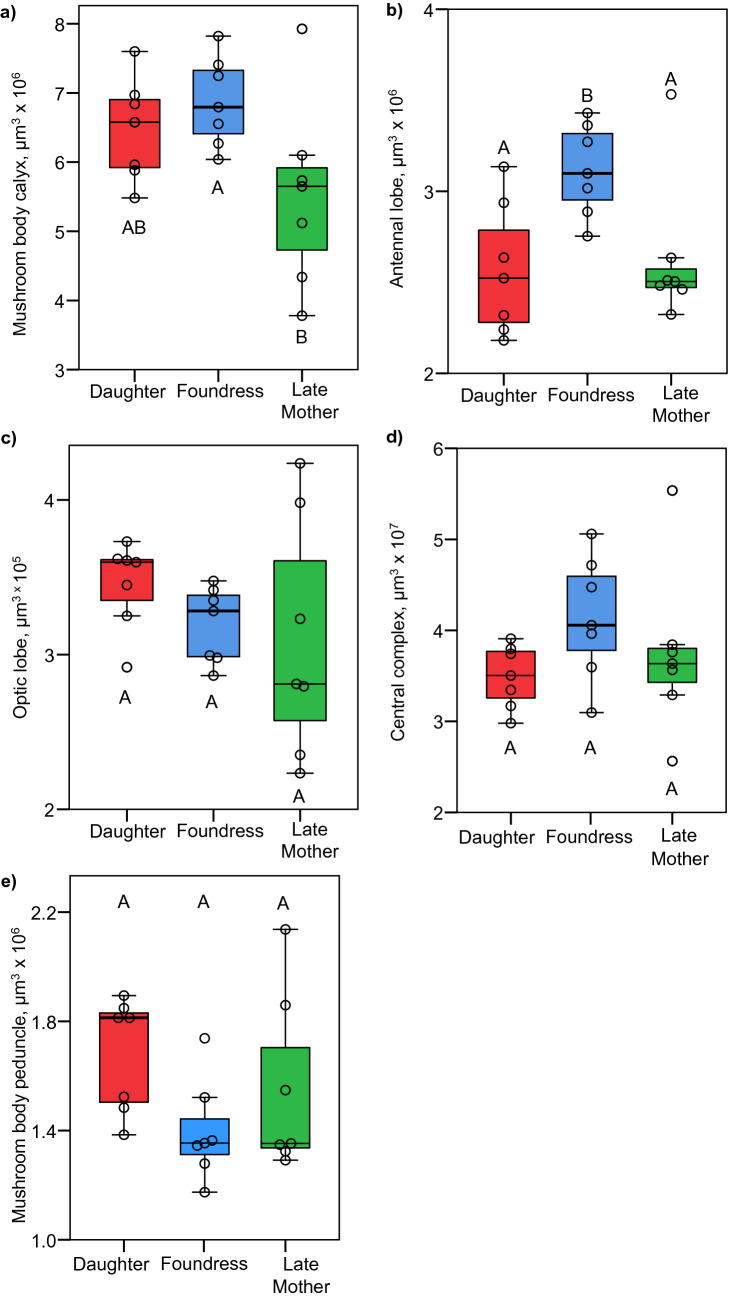
Size-corrected volumes of the mushroom body calyces (**a**), Antennal lobes (**b**), optic lobes (**c**), central complex (**d**), and MB peduncle (**f**) for each group (N = 7 for each group). Groups that do not share a letter are significantly different from each other based on an ANOVA followed by Tukey’s HSD pairwise comparisons.

Our predictions for enlargement of the MB calyx, OL, and AL were not met. Late mothers had smaller MB calyces than foundresses (*F*_2,18_ = 3.691, *p* = 0.045; Tukey’s post-hoc *p* = 0.041; Fig. 3a). There were no other significant pairwise differences in MB calyx volume (Fig. 3a). Both late mothers and newly emerged daughters had smaller antennal lobes than foundresses (*F*_2,18_ = 5.543, *p* = 0.013; Fig. 3b; Tukey’s post-hoc *p* = 0.040 and 0.018, respectively). There were no significant differences in optic lobe volume among groups (*F*_2,18_ = 0.994, *p* = 0.389; Fig. 3c). There were no significant differences in whole brain size (*F*_2,18_ = 2.296, *p* = 0.124), nor in the size of the central complex (*F*_2,18_ = 1.596, *p* = 0.230; Fig. 3d) or the MB peduncle (*F*_2,18_ = 2.313, *p* = 0.128; Fig. 3e) among groups.

### Interactions between physical and neurobiological measures

**Figure 4:**
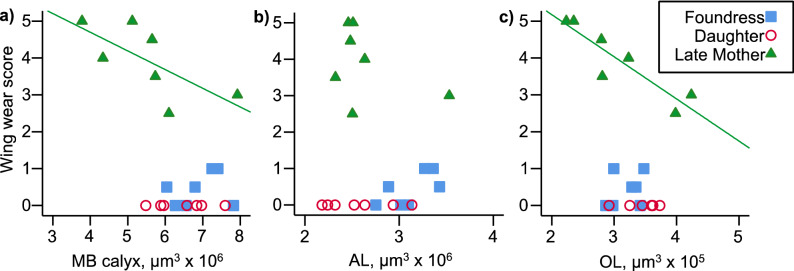
Degree of wing wear plotted against the volume of the MB calyx (**a**), AL (**b**), and OL (**c**). In all panels, late mothers are represented by green filled triangles, foundresses by blue filled squares, and newly emerged daughters by open red circles. Regression lines are shown for statistically significant within-group relationships (Spearman’s rank correlation) only, and represent linear regressions (N = 7 for each group).

Wing wear, a correlate of foraging experience, negatively correlated with MB calyx volume (rho = − 0.43, p = 0.049; Fig. 4a). This relationship is driven entirely by the late mothers group (rho = − 0.83, N = 7, p = 0.021). There is no correlation between wing wear and MB calyx volume among the foundresses (rho = 0.23, N = 7, p = 0.625); newly emerged daughters showed no wing wear so were not analyzed separately. Antennal lobe volume did not correlate with wing wear, whether all bees were analyzed together (rho = − 0.06, p = 0.801), or separately by group (late mothers rho = − 0.23, N = 7, p = 0.613; foundress rho = 0.61, N = 7, p = 0.150; Fig. 4b). Optic lobe volume did not correlate with wing wear when all bees were analyzed together (rho = − 0.41, p = 0.069). However, wing wear strongly and negatively correlated with OL volume among the late mothers (rho =− 0.92, N = 7, p = 0.003), but not the foundresses (rho = 0.44, N = 7, p = 0.330; Fig. 4c). Whole brain volume did not significantly correlate with wing wear for all bees (rho =− 0.14, p = 0.552) or the foundress and late mothers analyzed separately (foundresses: rho = 0.23, N = 7, p = 0.625; late mothers: rho =− 0.60, N = 7, p = 0.159). There was no correlation between CX and wing wear (rho = 0.08, p = 0.744), nor MB peduncle and wing wear (rho = − 0.17, p = 0.475).

**Figure 5:**
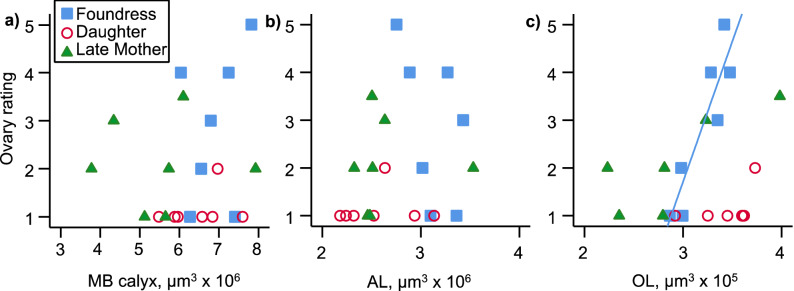
Ovary development plotted against the volume of the MB calyx (**a**), AL (**b**), and OL (**c**). In all panels, late mothers are represented by green filled triangles, foundresses by blue filled squares, and newly emerged daughters by open red circles. Regression lines are shown for statistically significant within-group relationships (Spearman’s rank correlation) only, and represent linear regressions (N = 7 for each group).

**Figure 6:**
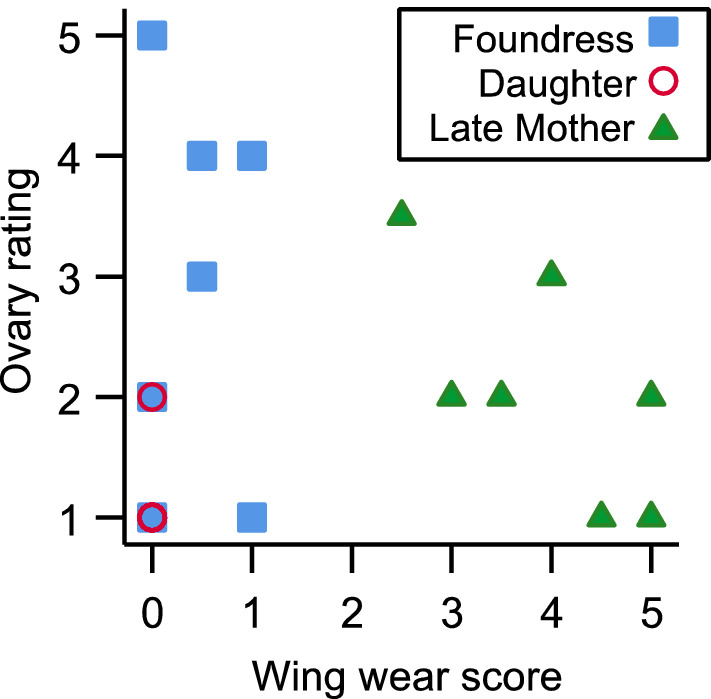
Ovary development plotted against wing wear; late mothers are represented by green filled triangles, foundresses by blue filled squares, and newly emerged daughters by open red circles. Note that six newly emerged daughter individuals are clustered at x = 0, y = 1. N = 7 for each group.

Ovary development did not correlate with MB calyx volume for all bees in the study analyzed together (rho = 0.17, p = 0.173), or separately by group (late mothers rho = 0.17, N = 7, p = 0.168; foundress rho = 0.26, N = 7, p = 0.582; only one newly emerged daughter showed any ovarian development, so we did not calculate correlations for this group, Fig. 5a). Likewise, AL volume did not correlate with ovary development for all bees in the study (rho = 0.29, p = 0.202) or individual groups (late mothers rho = 0.43, N = 7, p = 0.335; foundress rho = − 0.53, N = 7, p = 0.224; Fig. 5b). OL volume did not correlate with ovary development across all bees (rho = − 0.08, p = 0.740), or late mothers analyzed alone (rho = 0.58, N = 7, p = 0.172) but there was a strong correlation within the foundress group (rho = 0.82, N = 7, p = 0.024; Fig. 5c). There was no correlation between CX and ovary size (rho = 0.28, p = 0.225) nor MB peduncle and ovary size (rho = − 0.07, p = 0.763). Ovary development did not correlate with wing wear (rho = 0.24, p = 0.304), although a non-significant negative trend was seen in late mothers (rho = − 0.67, N = 7, p = 0.099; Fig. 6).

## Discussion

Here we show that neural investment in the MB calyces and AL, but not the OL, declines in advanced age in the subsocial bee *Ceratina calcarata*. This is contrary to what we predicted based on previous studies on the effect of experience on the insect brain. However, most previous studies of experience on insect brains have not followed individuals well past the onset of foraging, and those that did focused on the non-reproductive workers of eusocial ants or bees. Below, we first describe our ovary size and wing wear data in terms of reproductive development and foraging behavior. We then interpret our brain volume data in terms of the bees’ life history. We next discuss how our data comparing newly emerged daughters and reproductive foundresses are consistent with previous studies of the influence of experience on brain development. We then explain how our data showing a decline in MB calyx and AL volume in the late mothers relative to foundresses are not consistent with previous studies. Lastly, we contrast the influence of social experience in *C. calcarata* with *C. australensis* and outline directions for future study.

### Ovary size and wing wear

Females in this species emerge as adults in late summer (August) before entering diapause, after which they initiate a new nest and forage to provision offspring the following spring, continuing into the summer^34^. Our ovarian development measurements show that late summer, recently emerged daughters have little ovarian development, as expected. However, foundresses, which are actively provisioning nests, have enlarged ovaries. The large variability in foundress ovarian development of this group may arise from some bees being collected before fully enlarging their ovaries, while others were collected upon reproductive maturity^44^. For example, a study of the sweat bee *Megalopta genalis* showed small ovaries in newly emerged bees and large ovaries in established reproductives, but wide variation in the ovaries of foundresses^45^. The lack of wing wear in some *C. calcarata* foundress females in this study supports the interpretation that some foundresses have only just begun foraging, as wing wear generally correlates with flight activity^34,35^. By late summer, females have ceased reproduction and provisioning, and as a result ovarian development regresses, as reflected in the lower mean ovary rating of late summer mothers, and the negative, but non-significant, correlation between wing wear and ovary development.

### Life history and brain development

Our brain measurements suggest that after reproduction and initial foraging have ceased, *C. calcarata* females reduce, or cease to maintain, neural investment in the MB calyces and AL. The size-corrected volume of both the MB calyces and AL are smaller in late mothers than in foundresses; there were no significant differences in OL volume. Examination of the correlations between wing wear, which is a correlate of lifetime foraging activity, and neural investment further support the interpretation of reduced neural investment as females become post-reproductive. Among the late mothers, those with the most worn wings also had the smallest MB calyces and OLs. Interestingly, all three brain areas we studied (MB calyces, AL, OL) showed a negative correlation with wing wear in the late mothers (although the AL correlation was not significant), while all three brain areas showed a positive correlation with wing wear in foundresses (but none of these correlations were significant). This might indicate that experience may lead to increased neural investment in foundresses, who are enlarging their ovaries, initiating nests, and provisioning offspring. However, in late-summer mothers, experience, as indicated by wing wear, may instead be associated with post-reproductive senescence and neural decline. An alternative hypothesis is that foundresses, but not late mothers, require higher investment in MB calyces and ALs for initial larval provisioning that then regresses in late mothers. Further investigation, such as experiments in which the experience of foundresses and late-summer mothers is controlled [e.g.^5,11,15,19,30^] and observations are extended throughout the reproductive season [e.g.^7,32^] is required to test this hypotheses.

The typical pattern seen in adult insects is for MB and sensory neuropil volume to increase with age and foraging experience^4–20,32^. Studies that disentangle the two factors show that neuropil growth often occurs independent of experience at the beginning of adult life, after which accumulated sensory experience leads to further volume expansion^5,11,15,19,30,32^. In our study, AL volume shows a pattern of increased investment in foundresses relative to newly emerged daughters, which suggests a combined effect of age and experience, but MB calyx and OL volumes do not differ between these two groups. However, the positive, albeit non-significant, wing-wear correlations among foundresses might indicate that neuropil may generally increase with experience as females begin reproducing. Further studies examining newly emerged daughter brains immediately after adult emergence and experimentally manipulating the foraging experience of foundresses (e.g.^19^) are required to determine to what extent brain development between the newly emerged daughter and foundress stages is age vs. experience dependent. While our study was not designed to explicitly test for experience-expectant plasticity, this result is consistent with the two other studies of solitary bees which showed no experience-expectant plasticity^19,23^.

### Post-reproductive reduction of MB calyx and AL volume

The most dramatic finding of our study, that MB and AL volumes decreased from the foundress to the post-reproductive, late mother stage, is not typical of other insects studied. In two species of harvester ant queens, total brain volume, optic lobe volume, and, in one species, MB calyx volume, decreased after the foundress phase^46^. In the brains of foragers that became replacement reproductives in the queenless ant, *Harpegnathos saltator*, all measured brain areas showed decreased volume^47,48^. In both studies, the results were interpreted in terms of the queens reducing the metabolic maintenance costs of expensive neural tissue when they required less neural investment after establishing a nest underground with workers to forage and care for the brood. Once the queens had workers, they no longer required the navigation ability to forage, nor received the visual stimuli of doing so^46,47^. If *C. calcarata* females no longer require as much brain tissue after reproducing and rearing offspring to adulthood, they may similarly reduce investment in neural tissue. However, unlike ant foundress-turned-queens, *C. calcarata* late mothers still need to forage. They provide their newly emerged daughters with additional nutrition that increases their chances of surviving the following winter^35^. However, late mothers are near the end of their life: in a previous study, 93% died by September^35^.

In other species studied, age- and experience-related enlargement of the MB or other areas of the brain does not revert. In honeybee workers that are overwintering (and thus not foraging) there is no reduction in MB volume^33^. Likewise, in honeybee foragers that were manipulated to revert back to nursing behavior (workers typically nurse first, then forage), there was no reduction of MB volume back to lower, nurse-typical levels^33^. Previous studies have found that while ants and honeybees may show behavioral and physiological signs of senescence, there are not volumetric changes in the brain associated with senescence^49,50^. Workers of the ant *Camponotus floridanus* even showed continued MB volume increases up to six months of age^7,32^. However, all of these studies cited above were conducted on workers of eusocial species, which are not post-reproductive, because workers do not reproduce. Workers instead accrue indirect fitness by helping the colony reproduce throughout their lives. The only studies to examine the development of brain structures of solitary or small-colony bees either used individuals still engaged in reproduction^15,18,23,28^ or tropical species with no distinct end to the reproductive season^27,31^. Likewise, studies demonstrating experience-based enlargement of the MB in Lepidoptera also did not include post-reproductive individuals^13,14,51^. In *Drosophila,* changes in individual neurons and number of synapses, but not overall MB volume, are associated with senescence^52^. We hypothesize that *C. calcarata* life history may prioritize reproduction, resulting in reduced investment in neural structures late in life. Further studies of other solitary insects are required to test this hypothesis.

### Effects of social interactions

In the only other species of *Ceratina* bees studied to date, *C. australensis,* reproductive females showed experience dependent expansion of the MB calyces, including a positive correlation of both wing wear and ovarian development with MB calyx volume^15^. In our study, we found little effect of ovarian development on *C. calcarata* brain structure, except for a correlation between OL volume and ovary development in foundresses. In another interesting difference between the two *Ceratina* species, *C. australensis* showed additional MB calyx enlargement associated with social dominance (in *C. australensis,* some females nest with a subordinate sister). This is opposite to what we found in *C. calcarata,* where late summer mothers with newly emerged daughters in their nest had smaller MBs than early season foundresses. This suggests that the nature of sociality in the two species (semisocial, with a dominance hierarchy between sisters in *C. australensis,* and subsocial, with maternal care of daughters in the nest in *C. calcarata*) imposes fundamentally different cognitive demands. Given that both species have sequenced genomes^53,54^, are in separate subgenera of a genus with a well-established phylogeny^55,56^, exhibit a range of social behaviors and are experimentally tractable^57–60^, *Ceratina* bees could be a productive system for studying the effects of development, reproduction, and social interactions on neural investment.

## Data Availability

The data that support the findings of this study are openly available in figshare.com https://doi.org/10.6084/m9.figshare.19620615.
